# Postpartum Mothers’ Mental Health in a Conflict-Affected Region: A Cross-Sectional Study of Emotion Regulation and Social Support

**DOI:** 10.3390/jcm14041244

**Published:** 2025-02-13

**Authors:** Shirly Mor, Yaron Sela, Shahar Lev-Ari

**Affiliations:** 1. Department of Health Promotion, School of Public Health, Faculty of Medicine, Tel-Aviv University, Tel-Aviv 6997801, Israel; morshirly@mail.tau.ac.il (S.M.); yaron@pvalue.co.il (Y.S.); 2. Department of Genetics, School of Medicine, Stanford University, Stanford, CA 94305, USA

**Keywords:** postpartum mothers, mental health, emotion regulation, social support, promoting mental health, childbirth, mental health during conflict

## Abstract

**Background:** The transition through pregnancy, childbirth, and postpartum significantly impacts maternal mental health, influencing both individual and family well-being. While social support and emotion regulation serve as protective factors generally, their role and impact during periods of conflict remains understudied. **Methods:** We conducted a cross-sectional study of 400 Jewish mothers (up to two years postpartum) from a representative sample in Israel during a period of conflict. Participants were recruited through the Sekernet platform, a validated online survey tool in Israel. The study population included Jewish mothers up to two years postpartum, aged 18–45, without a history of diagnosed mental health disorders. Inclusion criteria specified mothers aged 18–45 and within two years postpartum, while exclusion criteria included mothers under 18, over 45, more than two years postpartum, or with a history of diagnosed mental illness or psychiatric disorders. Using validated instruments, we assessed psychological well-being (PWB), anxiety (GAD-7), perceived stress (PSS), resilience (CD-RISC), emotion regulation strategies (ERQ), quality of life (WHO-5), social support (MSPSS), and post-traumatic stress symptoms (PCL-5). Additionally, exposure to conflict-related media and direct exposure to war events were measured using self-reported questionnaires evaluating frequency and type of exposure during the conflict period. **Results:** Cognitive reappraisal and resilience positively correlated with psychological well-being (*p* < 0.01), while expressive suppression and general stress negatively correlated with both psychological well-being and quality of life (*p* < 0.01). Mediation analysis revealed that social support significantly mediated the effects of stress on psychological well-being (β = −0.060; *p* < 0.05) and quality of life (β = −0.05; *p* < 0.05). Additionally, exposure to conflict-related media and post-traumatic stress symptoms correlated with reduced well-being and increased anxiety. **Conclusions:** Our findings emphasize the vital roles of social support systems and adaptive emotional regulation strategies during the postpartum period, particularly in conflict settings. Healthcare providers should implement interventions that strengthen social support networks and teach adaptive emotion regulation skills to postpartum mothers in conflict zones.

## 1. Introduction

### 1.1. Promotion Mental Health Among Postpartum Mothers

The postpartum period represents a critical window for maternal mental health that significantly impacts both mother and child outcomes. During this sensitive time, mothers face unique challenges that can affect their psychological well-being and capacity for infant care. Research has consistently demonstrated that maternal stress during and after pregnancy can adversely affect fetal development, leading to increased risks of premature labor, low birth weight, and impaired cognitive and emotional development in children [1,2]. The postpartum period was previously defined as extending up to two years after birth, to capture the prolonged psychological and social complexities faced by mothers during this time. These include adjustments to parenting roles, work–life balance, and family dynamics, which often persist beyond the traditional postpartum window [3,4].

In addition to these developmental risks, maternal stress has also been linked to epigenetic modifications that may have long-term intergenerational effects. DNA methylation, one of the primary epigenetic mechanisms, plays a critical role in regulating gene expression, particularly in genes related to stress and anxiety responses, such as GABBR1. While most studies focus on prenatal stress, the postpartum period may similarly influence epigenetic pathways, further emphasizing the importance of maternal mental health interventions during this stage [5]. The prevalence of anxiety among postpartum women ranges from 12.2% to 39%, markedly higher than rates observed in the general population [3]. The economic impact is substantial—in the United States alone, postpartum depression results in 90% higher healthcare costs compared to non-depressed counterparts [6], while in the UK, the annual societal cost of depression, anxiety, and psychological treatments per birth cohort reaches approximately GBP 8.1 billion [7].

Given these challenges, the perinatal period presents a crucial opportunity for interventions aimed at building resilience and enhancing mental health [4]. Recent trends emphasize non-pharmacological approaches, reflecting many women’s preference for emotional support over medication, due to concerns about drug safety and dependency during pregnancy and breastfeeding [8,9]. These interventions focus on developing essential social and emotional skills, including self-esteem, self-efficacy, and coping abilities, which form the foundation for promoting psychological well-being and improving overall mental health.

Social support has emerged as a critical protective factor for postpartum mothers, with recent academic studies documenting its vital role in improving mental health outcomes. Research demonstrates that social support significantly buffers against postpartum depression while enhancing both psychological well-being and overall quality of life. Studies conducted during the COVID-19 pandemic highlighted how disruptions in social support networks adversely affected postpartum mental health, underscoring the necessity of robust support systems [10]. Research from Canada further reinforces these findings, showing that mothers with stronger post-birth support networks demonstrated a significantly lower risk of developing postpartum depression [11].

Emotion regulation, particularly through cognitive reappraisal, has proven beneficial for postpartum mothers by enabling them to reinterpret stressful situations and reduce their emotional impact. This strategy aids in managing negative emotions and enhances overall mental health during the postpartum period. Recent findings from emotion regulation research suggest that effective strategies like cognitive reappraisal not only help reduce personal distress but also foster better social interactions, which can lead to increased social support [10,11]. 

According to the International Union for Health Promotion and [12], mental health interventions should prioritize the social and emotional development of infants and mothers. Effective strategies include enhanced prenatal care, home visits, and comprehensive parenting programs, all aimed at fostering positive mental health from early childhood.

Mothers have the potential to increase their personal maturity and promote their mental health in a positive way. Therefore, the challenge facing public health today is to achieve a better understanding of the mechanisms that enable women to develop and maintain positive mental health in the postpartum period and to understand how these mechanisms are preserved over time.

### 1.2. Postpartum Mothers’ Mental Health During Conflict 

The impact of conflict on postpartum mothers presents unique challenges that extend beyond typical postpartum adaptation. War and conflict situations create an environment of heightened stress, insecurity, and trauma that can profoundly influence women’s experiences during the postpartum period.

In October 2023, Israel experienced a period of intense conflict marked by widespread security threats and civilian casualties, creating significant disruptions to daily life. The conflict resulted in heightened levels of anxiety, grief, and insecurity, particularly among vulnerable populations such as postpartum mothers. The combination of direct exposure to traumatic events, such as sirens and attacks, and indirect stressors, including media coverage and community disruptions, further compounded the challenges faced by this group during an already sensitive period of life [13].

These circumstances affect multiple dimensions of maternal well-being. Physical impacts include compromised access to essential healthcare services, leading to inadequate prenatal care, an increased risk of childbirth complications, and limited postnatal support [14]. Infrastructure disruption and displacement further complicate access to medical facilities, supplies, and skilled healthcare professionals [15]. These challenges contribute to elevated maternal mortality rates and pregnancy-related complications in conflict-affected regions [14].

Psychological effects manifest through increased trauma exposure and chronic stress. Women face heightened risks of anxiety, depression, and post-traumatic stress disorder during the postpartum period [16]. The pervasive uncertainty and fear associated with conflict zones can intensify feelings of helplessness and despair, complicating the already challenging demands of early parenthood [16,17].

Social impacts include the disruption of traditional support networks and community structures crucial for maternal well-being. Displacement, family separation, and the loss of social connections can exacerbate isolation during the postpartum period [15]. The breakdown of community cohesion can impede access to social support, childcare assistance, and essential resources for maternal and infant care [14].

Given these multifaceted challenges, understanding how different factors interact to influence maternal mental health during conflict becomes crucial [17]. This study aims to examine the relationships between psychological well-being, quality of life, stress, anxiety, resilience, and emotional regulation skills among mothers up to two years postpartum during conflict. By analyzing these relationships and their mediating factors, we seek to identify potential intervention points for supporting maternal mental health in conflict settings.

The primary objective of this study is to examine the relationships between maternal psychological well-being, quality of life, emotion regulation strategies, resilience, social support, perceived stress, anxiety, post-traumatic stress symptoms, and exposure to conflict-related media during the postpartum period in a conflict-affected region. Specifically, we aim to identify key predictors and mediators influencing maternal well-being and quality of life.

We hypothesize that adaptive emotion regulation strategies (e.g., cognitive reappraisal), higher levels of social support, resilience, and lower exposure to conflict-related media will be positively associated with psychological well-being and quality of life. Conversely, maladaptive strategies (e.g., expressive suppression), higher levels of stress, anxiety, and post-traumatic stress symptoms will be negatively associated. Additionally, social support, time since delivery, and exposure to conflict-related media are hypothesized to mediate these relationships.

## 2. Methods

### 2.1. Study Design and Participants 

We conducted a cross-sectional analytical study in Israel targeting mothers up to two years postpartum, inviting the participants using the Sekernet platform (sekernet.co.il). Sekernet is a leading online survey platform in Israel, recognized for its rigorous data quality standards. The platform employs multi-stage participant verification processes, strict data monitoring, and validation mechanisms, ensuring the reliability and integrity of the final dataset. The data were collected between December 2023 and January 2024, approximately three months after the onset of the war. This timeline was chosen to capture both immediate and prolonged impacts of the conflict on postpartum mothers. The study protocol was approved by the Ethics Committee (Institutional Review Board) of Tel Aviv University (#0008535-2; approval date: 2023-12-21). Eligible participants were aged 18-45 years at the time of birth, had no history of mental illness, and were residents of Israel. Participant recruitment occurred through online panels and social media networks. To ensure representativeness, we stratified the sample proportionally according to population size across Israel’s main districts and maternal age distribution at birth. This study was conducted in accordance with the STROBE guidelines for reporting cross-sectional studies to ensure transparency and adherence to rigorous reporting standards. 

### 2.2. Measures

*Psychological Well-being (PWB):* In assessing psychological well-being (PWB), we employed the short version of the scale comprising 18 items [18,19]. Respondents were instructed to indicate the extent of their agreement or disagreement with each statement on a 7-point Likert scale (1 = strongly agree; 7 = strongly disagree). The overall reliability of the scale in the current study was deemed acceptable (α = 0.80).

*General Anxiety Disorder (GAD-7):* The Generalized Anxiety Disorder 7-Item Scale [20] was used. In this questionnaire, respondents rate how often they were bothered by each of the problems listed in the statements using a 4-point scale (0 = never; 3 = almost every day). A score above 8 indicates the likelihood of an anxiety disorder. The general reliability of the general scale in the present study was good (α = 0.92).

*Emotion Regulation Questionnaire (ERQ).* The Emotion Regulation Questionnaire (ERQ) utilized in this study, as developed by Gross and John [21], assesses emotional regulation through a 7-point Likert scale (1 = strongly agree; 7 = strongly disagree). It measures two aspects: cognitive reappraisal (α = 0.88) and expressive suppression (α = 0.82), with higher scores indicating greater emotional regulation capability.

*Perceived Stress Scale (PSS):* The Perceived Stress Scale (PSS), adapted from Cohen, Kamarck, and Mermelstein [22], evaluates respondents’ stress levels on a 5-point scale (0 = never; 4 = very often), with higher scores indicating increased stress levels (α = 0.88).

*Resilience (CD-RISC*). Resilience was assessed using the Connor–Davidson Resilience Scale (10-item CD-RISC) by Connor and Davidson [23], employing a 5-point scale (0 = not at all; 4 = very much), with an acceptable reliability (α = 0.71). 

*Quality of life (WHO-5).* Quality of life was measured using the psychological general well-being scale by the WHO-5 [24], scored on a 6-point scale (0 = never; 5 = all the time), with higher scores indicating better quality of life (α = 0.89).

*Social support (MSPSS).* We used the Multidimensional Scale of Perceived Social Support (MSPSS) [25]. In this questionnaire, the respondents rate to what extent they agree or disagree with the statements using a 7-point scale (1 = very strongly disagree; 7 = very strongly agree). The questionnaire measures three dimensions of social support: support from friends (α = 0.94), family support (α = 0.92), and support from another significant factor (α = 0.93). Higher scores mean higher levels of psychological well-being. The general reliability of the general scale in the present study was good (α = 0.94).

### 2.3. Data Analysis

Data analysis was conducted using the Statistical Package for the Social Sciences (SPSS), version 26. We produced means and standard deviations of the main study variables. Pearson correlation analyses were performed to explore the bi-variate relationships between the main study variables (e.g., psychological well-being, quality of life, general stress, social support, and emotional regulation strategies).

We used multiple linear regression analyses to assess the direct CIs of emotional regulation strategies (reappraisal and expressive suppression), resilience, and stress on psychological well-being and quality of life. These models were adjusted for potential confounders, including demographic variables like age, educational level, and socioeconomic status.

In order to examine the research model, a path analysis using structural equations (SEM) was conducted, which evaluated relationship estimates between the variables. In addition, goodness of fit indices were produced: a goodness of fit index (GFI), a comparative fit index (CFI) (both with a value above 0.9 for a good fit), and a root mean square error of approximation index (RMSEA), which was expected to be 0.08 or lower. In the model, a statistical control was performed on the demographic variables and the war variables that were found to be related to the dependent variables (quality of life and psychological well-being). The significance level for all tests was set at *p* < 0.05.

## 3. Results

### 3.1. Participant Characteristics

Our sample comprised 400 participants ranging in age from 20 to 45 years (M = 30.85; SD = 5.51). Religious affiliation was distributed across secular (32.9%), traditional (24.9%), religious (17.7%), and ultra-Orthodox (24.4%) categories. The vast majority of participants were married (89.0%), with smaller proportions being single (9.2%) or divorced (0.2%). Educational attainment was relatively high, with 40.0% holding Bachelor’s degrees, 27.0% completing certificate studies, and 18.3% holding Master’s degrees. Regarding employment, 60.2% worked full-time, 16.3% worked part-time, and 14.5% were students. Demographic and birth information is presented in Table 1.

#### 3.1.1. Inter-Correlations of Main Study Variables

Table 2 presents the descriptive statistics and Pearson correlations between main study variables. The results showed that general psychological well-being (GPW) was positively correlated with social support (r = 0.55; *p* < 0.001) and quality of life (QoL) (r = 0.50; *p* < 0.001), and negatively correlated with general anxiety (r = −0.42; *p* < 0.001) and stress (r = −0.49; *p* < 0.001). Reappraisal strategies were highly correlated with emotional regulation (r = 0.80; *p* < 0.001), while expressive suppression was moderately correlated with emotional regulation (r = 0.71; *p* < 0.001) and negatively correlated with GPW (r = −0.41; *p* < 0.001).

Additionally, general anxiety was strongly positively correlated with stress (r = 0.73; *p* < 0.001), indicating a robust relationship between higher anxiety levels and increased stress. Resilience showed negative correlations with both general anxiety (r = −0.24; *p* < 0.001) and stress (r = −0.40; *p* < 0.001), and a positive relationship with social support (r = 0.23; *p* < 0.001) and quality of life (r = 0.37; *p* < 0.001), emphasizing its protective role against negative psychological states and its association with better life outcomes.

Social support was also negatively correlated with stress (r = −0.42; *p* < 0.001) and positively associated with quality of life (r = 0.43; *p* < 0.001). These findings highlight the importance of social support and emotional regulation in enhancing well-being and mitigating stress and anxiety.

#### 3.1.2. Correlations Between War-Related Variables and Main Study Variables

Table 3 presents the Pearson correlations between war-related variables and main study variables. The results showed that general psychological well-being showed significant negative correlations with various media exposures and PTSD. Specifically, well-being was negatively correlated with watching news during the first week (r = −0.14; *p* < 0.001), watching news in the last two weeks (r = −0.13; *p* < 0.001), exposure to videos (r = −0.22; *p* < 0.001), and PTSD (r = −0.27; *p* < 0.001). These findings suggest that increased consumption of war-related media and higher levels of PTSD symptoms are associated with lower psychological well-being. General anxiety presented a complex pattern of correlations. It was inversely correlated with exposure to war events (r = −0.11; *p* = 0.021) but positively correlated with media consumption and PTSD. Specifically, anxiety was positively correlated with watching news in the first week (r = 0.21; *p* < 0.001), watching news in the last two weeks (r = 0.12; *p* = 0.02), exposure to videos (r = 0.24; *p* < 0.001), and PTSD (r = 0.52; *p* < 0.001). This indicates that anxiety levels increase with greater media exposure and higher PTSD symptoms. Expressive suppression showed a slight positive correlation with watching news in the last two weeks (r = 0.12; *p* = 0.020) and PTSD (r = 0.11; *p* = 0.030). This suggests that higher use of suppression as a coping mechanism mildly correlates with increased exposure to recent news and PTSD symptoms. General stress was negatively correlated with exposure to war events (r = −0.11; *p* = 0.033). However, it was positively correlated with watching news in the first week (r = 0.23; *p* < 0.001), exposure to videos (r = 0.25; *p* < 0.001), and PTSD (r = 0.45; *p* < 0.001). These findings highlight that stress levels are particularly sensitive to recent media exposures rather than initial events. Resilience demonstrated a slight positive correlation with exposure to war events (r = 0.06) but was negatively correlated with exposure to videos (r = −0.13; *p* = 0.006) and PTSD (r = −0.23; *p* < 0.001). This indicates that resilience may slightly improve with direct war exposure but deteriorates with video exposure and higher PTSD symptoms. Social support was negatively correlated with PTSD (r = −0.12; *p* = 0.0120), underscoring the protective role of social resources against PTSD symptoms. Quality of life was positively correlated with exposure to war events (r = 0.08). However, it showed strong negative correlations with watching news in the first week (r = −0.24; *p* < 0.001), watching news in the last two weeks (r = −0.17; *p* < 0.001), exposure to videos (r = −0.30; *p* < 0.001), and PTSD (r = −0.40; *p* < 0.001). These results suggest that quality of life deteriorates with increasing consumption of war-related media and the presence of PTSD symptoms.

#### 3.1.3. Multivariate Models Predicting General Psychological Well-Being and Quality of Life

Table 4 presents multivariate linear regressions predicting general psychological well-being and quality of life.

The results of the model predicting general psychological well-being, showed that after controlling the sociodemographic variables, regulation through reappraisal showed a positive effect (β = 0.201; *p* < 0.001), while expressive suppression was a negative predictor (β = −0.283; *p* < 0.001). Social support also had a strong positive association with well−being (β = 0.256; *p* <0.001), underscoring the importance of social networks. 

The results of the model predicting quality of life showed that social support (β = 0.200; *p* < 0.001) was a positive predictor. However, general stress (β = −0.466; *p* < 0.001) and exposure to news in the last two weeks (β = −0.112; *p* = 0.006) negatively predicted quality of life.

#### 3.1.4. Path Analysis Model of Social Supports Mediates the Relationship of Anxiety, Emotional Regulation, Stress, and Resilience with General Psychological Well-Being and Quality of Life

The model demonstrated a good fit (χ^2^(5) = 47.89; *p* = 0.001; GFI = 0.97; CFI = 0.95; NFI = 0.95; RMSEA = 0.14). Direct relationships were examined between independent variables and psychological well-being, revealing that reappraisal (β = 0.18; *p* < 0.001) and resilience (β = 0.17; *p* < 0.001) positively influence psychological well-being, while expressive suppression (β = −0.30; *p* < 0.001) and general stress (β = −0.13; *p* = 0.031) negatively impact it.

For quality of life, general stress was a significant negative predictor (β = −0.452; *p* < 0.001). Regarding social support, general stress (β = −0.240; *p* < 0.001) and expressive suppression (β = −0.314; *p* < 0.001) were negatively related, whereas reappraisal had a positive relationship (β = 0.131; *p* < 0.001). Higher social support correlated with increased psychological well-being (β = 0.238; *p* < 0.001) and quality of life (β = 0.168; *p* < 0.001).

**Figure 1:**
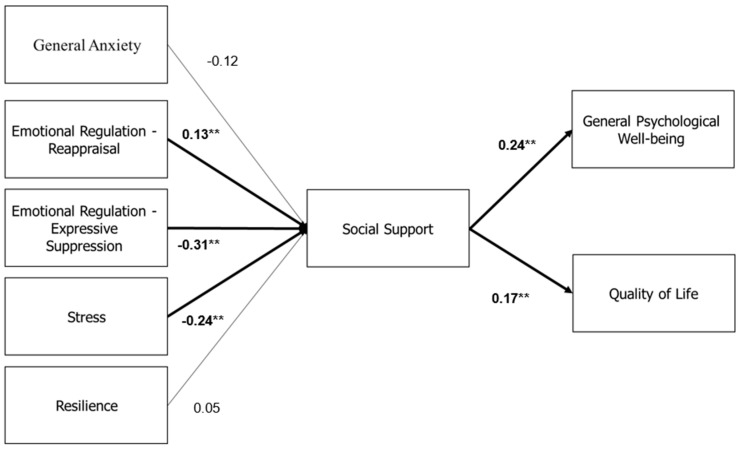
Social supports mediate the relationship of anxiety, emotional regulation, stress, and resilience with general psychological well-being and quality of life. The values in the figure represent beta coefficients; ** *p* < 0.01.

Mediation analysis indicated that social support mediated the relationship between general stress and both psychological well-being (β = −0.060; *p* = 0.010) and quality of life (β = −0.05; *p* = 0.120). Similarly, social support mediated the relationship between expressive suppression and psychological well-being (β = −0.07; *p* = 0.011) and quality of life (β = −0.05; *p* = 0.012). Conversely, social support enhanced the positive effects of reappraisal on psychological well-being (β = 0.03; *p* = 0.012) and quality of life (β = 0.02; *p* = 0.010). These findings suggest that adaptive emotional regulation strategies and strong social support systems are crucial for enhancing psychological well-being and quality of life in postpartum mothers, particularly in conflict settings (Figure 1).

## 4. Discussion

Our study provides significant insights into the factors influencing mental health among postpartum mothers, particularly emphasizing the interplay between social support, emotion regulation strategies, and psychological well-being during periods of conflict. The findings reveal several key mechanisms through which maternal mental health can be supported or compromised during this sensitive period.

The role of cognitive reappraisal emerges as particularly significant in our analysis. This emotion regulation strategy, which involves reinterpreting situations to alter their emotional impact, showed a strong positive association with psychological well-being. This finding aligns with Lazarus and Folkman’s cognitive appraisal theory [26], suggesting that helping mothers develop skills to reframe their experiences could effectively mitigate emotional challenges during the postpartum period. The practical implications of our finding maybe suggest that healthcare providers could incorporate reappraisal training into standard postpartum care protocols.

Expressive suppression, characterized by inhibiting emotional expression, demonstrated negative associations with psychological well-being. This result supports Gross’s process model of emotion regulation [27], which suggests that suppression may increase psychological distress by interfering with natural emotional processing and potentially exacerbating stress responses [28]. The finding indicates that postpartum mothers who habitually suppress their emotions may face increased risks for reduced psychological well-being, highlighting the need for interventions that encourage healthy emotional expression.

The relationship between general stress and psychological well-being emerged as an important factor in our analysis. The postpartum period inherently involves numerous stressors, from new caregiving responsibilities to physical recovery, and our findings indicate that these challenges are amplified in conflict situations. The negative relationship between stress and psychological well-being underscores the importance of stress management strategies in postpartum care [29], particularly in conflict-affected areas. Resilience emerged as a protective factor in our study. The positive relationship between resilience and psychological well-being aligns with the broader resilience framework, suggesting that higher resilience levels enable better coping with adversity [23]. For postpartum mothers facing both typical postpartum challenges and conflict-related stressors, fostering resilience appears crucial for maintaining and improving mental health [30]. While our findings indicate that expressive suppression was negatively associated with psychological well-being and quality of life, research suggests that in high-stress environments, such as conflict zones, it may serve as a short-term coping mechanism [31,32]. In contrast to Israeli culture, which combines individual expression with communal support, collectivist cultures, such as those in East Asia, often view expressive suppression as an adaptive strategy for maintaining social harmony and avoiding conflicts [32,33,34].

Our findings regarding social support provide compelling evidence for its role in maternal mental health. The strong positive relationships between social support and both psychological well-being and quality of life are aligned with previous research in postpartum populations [35]. The buffering hypothesis of social support appears especially relevant in our findings, as social support significantly mediated the relationship between stress and psychological outcomes [36]. This mediation effect suggests that social support can help mitigate the negative impacts of stress during the postpartum period [37], even in challenging conflict situations.

The conflict-specific aspects of our findings merit special attention. The negative correlations between media exposure and psychological well-being suggest that healthcare providers should consider providing guidance about media consumption during the postpartum period, particularly in conflict zones [16,17]. The relationship between PTSD symptoms and reduced well-being further emphasizes the need for trauma-informed care approaches in conflict-affected areas [15,38].

These findings carry significant practical implications for healthcare delivery. The integration of emotion regulation training into postpartum care, particularly focusing on cognitive reappraisal techniques, could provide mothers with valuable tools for managing emotional challenges [38]. Additionally, our results strongly support the development and maintenance of robust social support systems [39], suggesting that healthcare providers should actively facilitate community and peer connections to reduce isolation and stress among mothers.

### 4.1. Limitations and Future Directions 

Our study, while providing valuable insights into postpartum maternal mental health during conflict, has several limitations that warrant consideration. The cross-sectional nature of our research design limits our ability to establish causality in the relationships we observed. Although we found compelling associations between cognitive reappraisal, social support, and well-being, we cannot definitively determine whether improved emotion regulation leads to enhanced social support and well-being, or if women with better well-being and stronger social networks are naturally more inclined to use adaptive emotion regulation strategies. Future longitudinal research would help clarify these directional relationships and reveal how they evolve throughout the postpartum period.

Our reliance on self-report measures for assessing emotion regulation, social support, and psychological outcomes introduces potential measurement limitations. Participants may have overestimated their use of adaptive strategies like cognitive reappraisal or underreported their stress and depressive symptoms due to social desirability bias. Future research would benefit from incorporating more objective measures, such as physiological indicators of stress and well-being, or third-party assessments of social support and emotion regulation. Additionally, ecological momentary assessment methods could provide more accurate real-time data about emotional experiences and regulation strategies. The generalizability of our findings may be limited by the demographic characteristics of our sample. While we attempted to recruit a representative sample, our participants were predominantly from middle-to-upper socioeconomic backgrounds and had relatively high educational attainment. Cultural background, socioeconomic status, and the presence of a supportive partner can significantly influence both emotion regulation practices and access to social support. Future studies should prioritize including more diverse samples to examine how these findings apply across different cultural and social contexts, particularly in various conflict settings. Finally, we excluded participants with a history of mental illness. This criterion may narrow the scope of the findings and limit their applicability to mothers with a history of mental health disorders.

### 4.2. Concluding 

This study provides compelling evidence for the importance of emotion regulation strategies and social support in shaping the mental health and quality of life of postpartum mothers during conflict. Our findings demonstrate that cognitive reappraisal serves as a beneficial strategy for managing emotional challenges, while expressive suppression may have detrimental effects on psychological well-being. The mediating role of social support in the relationship between stress and well-being highlights the necessity of maintaining and strengthening social support networks during the postpartum period, particularly in conflict situations. 

These results may have implications for clinical practice and public health interventions. Healthcare providers should consider implementing comprehensive programs that integrate emotion regulation training into standard postpartum care, with particular emphasis on teaching cognitive reappraisal techniques. Additionally, healthcare systems should prioritize the development of structured social support networks for new mothers, especially in conflict-affected areas where traditional support systems may be disrupted.

Our findings regarding the negative impact of conflict-related media exposure suggest the need for specific guidance about media consumption during the postpartum period. Healthcare providers should consider developing guidelines for managing media exposure while maintaining necessary awareness of safety information in conflict zones.

The relationship between maternal mental health and conflict exposure revealed in our study also highlights the broader public health implications of conflict on maternal and child health. This understanding should inform both healthcare policy and peace-building initiatives, recognizing that supporting maternal mental health is crucial not only for individual well-being but also for community resilience in conflict-affected regions.

## Figures and Tables

**Table 1:** Demographic and birth information of the sample.

Variable	N	%	M	SD	Range
Age			30.85	5.51	20–45
Religious Affiliation					
Secular	132	32.9			
Traditional	100	24.9			
Religious	71	17.7			
Ultra-Orthodox	98	24.4			
Family Status					
Single	37	9.2			
Married	357	89.0			
Divorced	1	0.2			
Education					
No Formal Education	2	0.5			
Secondary Education	53	13.3			
Certificate Studies	108	27.0			
Bachelor’s Degree	160	40.0			
Master’s Degree	73	18.3			
Doctorate	4	0.9			
Current Employment Status					
Full-Time Employment	233	60.2			
Part-Time Employment	63	16.3			
Unemployed	28	7.2			
Student	56	14.5			
Birth Data					
Time Since Birth (in months)			10.72	6.70	0–24
Type of Birth					
Vaginal	308	76.8			
Instrumental	19	4.7			
Emergency Cesarean Section	40	10.0			
Planned Cesarean Section	27	6.7			
Other	7	1.7			
Entry Into Pregnancy					
Spontaneous (including insemination)	356	88.8			
IVF	45	11.2			
Duration of IVF (in months)			19.38	30.74	1–192
Is This the First Birth?	152	37.9			
Number of Births					
2	118	47.4			
3	69	27.7			
4	34	13.7			
5	12	4.8			
6	11	4.4			
7	5	2.0			
Number of Children Before the Last Birth			1.96	1.25	1–7

N—number, M—mean, SD—Standard Deviation, IVF—In Vitro Fertilization.

**Table 2:** Means, standard deviations, and Pearson correlations between main study variables (n = 400).

	M	SD	1	2	3	4	5	6	7	8
1. General Psychological Well-being	5.27	0.75								
2. General Anxiety	1.02	0.78	−0.042 **							
3. Emotional Regulation	4.41	1.02	−0.04	0.01						
4. Reappraisal	5.15	1.21	0.28 **	−0.15 **	0.80 **					
5. Expressive Suppression	3.31	1.52	−0.41 **	0.21 **	0.071 **	0.015 **				
6. Stress	1.97	0.71	−0.049 **	0.73 **	−0.08	−0.24 **	0.15 **			
7. Resilience	2.80	0.78	0.41 **	−0.24 **	0.17 **	0.28 **	−0.06	−0.40 **		
8. Social Support	5.78	1.24	0.55 **	−0.34 **	−0.09	0.17 **	−0.35 **	−0.42 **	0.23 **	
9. Quality of Life	2.38	1.05	0.50 **	−0.57 **	0.03	0.17 **	−0.16 **	−0.68 **	0.37 **	0.43 **

** *p* < 0.01.

**Table 3:** Associations between war-related variables and main study variables (n = 400).

Exposure to War Events	Watching News in the First Week	Watching News in the Last Two Weeks	Exposure to Videos	PTSD
General Psychological Well-being	0.077	−0.14 **	−0.13 **	−0.22 **
General Anxiety	−0.11 *	0.21 **	0.12 *	0.24 **
Emotional Regulation	−0.05	0.06	0.07	0.01
Regulation—Reappraisal	−0.04	0.01	0.01	−0.01
Regulation—Expressive Suppression	−0.04	0.01	0.12*	0.05
General Stress	−0.11 *	0.23 **	0.04	0.25 **
Resilience	0.06	−0.06	0.01	−0.13 *
Social Support	0.03	−0.01	0.05	−0.07
Quality of Life	0.08	−0.24 **	−0.17 **	−0.30 **

* *p* < 0.05, ** *p* < 0.01.; Post-Traumatic Stress Disorder—PTSD.

**Table 4:** Multivariate linear regressions predicting general psychological well-being and quality of life.

	General Psychological Well-Being (n = 353)				Quality of Life (n = 353)			
	β	B	95% CI (Lower Bound)	95% CI (Upper Bound)	β	B	95% CI (Lower Bound)	95% CI (Upper Bound)
Level of Religiosity	−0.013	−0.001	−0.057	0.054	0.104 *	0.093	0.016	0.170
Level of Education	0.130	0.097	0.037	0.158	0.005	0.019	−0.065	0.103
Income Level	−0.014	−0.004	−0.032	0.024	0.029	0.013	−0.026	0.053
Exposure to War Events	−0.014	−0.027	−0.142	0.087	−0.040	−0.090	−0.249	0.070
Watching News in the First Week	0.026	0.010	−0.028	0.049	−0.032	−0.019	−0.072	0.035
Watching News in the Last Two Weeks	−0.072	−0.048	−0.097	0.001	−0.112 *	−0.096	−0.164	−0.028
Exposure to Videos	−0.063	−0.066	−0.159	0.027	−0.008	−0.020	−0.150	0.109
PTSD	−0.052	−0.051	−0.133	0.032	−0.046	−0.063	−0.178	0.051
General Anxiety	−0.018	−0.020	−0.136	0.095	−0.032	−0.046	−0.207	0.115
Regulation—Reappraisal	0.201 **	0.120	0.070	0.170	−0.058	−0.033	−0.102	0.036
Regulation—Expressive Suppression	−0.283 **	−0.134	−0.174	−0.093	0.066	0.025	−0.031	0.082
General Stress	−0.093	−0.149	−0.276	−0.022	−0.466 **	−0.709	−0.885	−0.532
Resilience	0.172 **	0.172	0.094	0.250	0.065	0.111	0.003	0.220
Social Support	0.256 **	0.168	0.116	0.220	0.200 **	0.192	0.119	0.265
Explained Variance	52.3%				52.7%			
Model Significance	26.71 **				27.16 **			

* *p* < 0.05; ** *p* < 0.01. Beta—B.

## Data Availability

The data that support the findings of this study are available from the corresponding author, S.L.-A., upon reasonable request.

## References

[1] Dunkel Schetter C. (2011). Psychological Science on Pregnancy: Stress Processes, Biopsychosocial Models, and Emerging Research Issues. Annu. Rev. Psychol..

[2] Polanska K., Hanke W., Ronchetti R., van den Hazel P., Barreto M., Gruszfeld D., Jerzynska J., Koppe J.G. (2017). Environmental Tobacco Smoke Exposure and Children’s Health. Acta Paediatr..

[3] Goodman J.H., Chenausky K.L., Freeman M.P. (2014). Anxiety Disorders During Pregnancy: A Systematic Review. J. Clin. Psychiatry.

[4] Lever Taylor B., Cavanagh K., Strauss C. (2016). The effectiveness of mindfulness-based interventions in the perinatal period: A systematic review and meta-analysis. PLoS ONE.

[5] Meaney M.J. (2010). Epigenetics and the biological definition of gene × environment interactions. Child Dev..

[6] Dagher R.K., McGovern P.M., Dowd B.E., Gjerdingen D.K. (2012). Postpartum Depression and Health Services Expenditures Among Employed Women. J. Occup. Environ. Med..

[7] Bauer A., Parsonage M., Knapp M., Iemmi V., Adelaja B. (2014). Costs of Perinatal Mental Health Problems.

[8] Battle C.L., Salisbury A.L., Schofield C.A., Ortiz-Hernandez S. (2013). Perinatal Antidepressant Use: Understanding Women’s Preferences and Concerns. J. Psychiatr. Pract..

[9] NICE (2014). Antenatal and Postnatal Mental Health: Clinical Management and Service Guidance.

[10] White L.K., Kornfield S.L., Himes M.M., Forkpa M., Waller R., Njoroge W.F., Barzilay R., Chaiyachati B.H., Burris H.H., Duncan A.F. (2023). The impact of postpartum social support on postpartum mental health outcomes during the COVID-19 pandemic. Arch. Women’s Ment. Health.

[11] Kim T.H., Connolly J.A., Tamim H. (2014). The effect of social support around pregnancy on postpartum depression among Canadian teen mothers and adult mothers in the maternity experiences survey. BMC Pregnancy Childbirth.

[12] International Union for Health Promotion and Education (2021). Global Position Statement on Mental Health Promotion.

[13] Katsoty D., Greidinger M., Neria Y., Segev A., Lurie I. (2024). A prediction model of PTSD in the Israeli population in the aftermath of October 7th, 2023, terrorist attack and the Israel–Hamas war. Isr. J. Health Policy Res..

[14] Finch J. (2018). Conflict and Health: Challenges and Opportunities in War Zones. J. Confl. Health.

[15] Spiegel P., Checchi F., Colombo S., Paik E. (2010). Health-Care Needs of People Affected by Conflict: Future Trends and Changing Frameworks. Lancet.

[16] Rieder M., Choonara I. (2012). Toxic Stress: Effects on the Developing Child. BMJ.

[17] Miller K.E., Rasmussen A. (2010). War Exposure, Daily Stressors, and Mental Health in Conflict and Post-Conflict Settings: Bridging the Divide Between Trauma-Focused and Psychosocial Frameworks. Soc. Sci. Med..

[18] Ryff C.D., Singer B.H., Love G.D. (2010). Positive Health: Connecting Well-Being with Biology. Philos. Trans. R. Soc. Lond. B Biol. Sci..

[19] Ryff C.D., Keyes C.L.M. (1995). The Structure of Psychological Well-Being Revisited. J. Pers. Soc. Psychol..

[20] Löwe B., Decker O., Müller S., Brähler E., Schellberg D., Herzog W., Herzberg P.Y. (2008). Validation and standardization of the Generalized Anxiety Disorder Screener (GAD-7) in the general population. Med. Care.

[21] Gross J.J., John O.P. (2003). Individual Differences in Two Emotion Regulation Processes: Implications for Affect, Relationships, and Well-Being. J. Pers. Soc. Psychol..

[22] Cohen S., Kamarck T., Mermelstein R. (1994). A Global Measure of Perceived Stress. J. Health Soc. Behav..

[23] Connor K.M., Davidson J.R.T. (2003). Development of a New Resilience Scale: The Connor-Davidson Resilience Scale (CD-RISC). Depress. Anxiety.

[24] Bech P. (1999). Measuring the Dimension of Psychological General Well-being by the WHO-5. Qual. Life Res..

[25] Zimet G.D., Dahlem N.W., Zimet S.G., Farley G.K. (1988). The multidimensional scale of perceived social support. J. Personal. Assess..

[26] Lazarus R.S., Folkman S. (1984). Stress, Appraisal, and Coping.

[27] Gross J.J. (1998). The Emerging Field of Emotion Regulation: An Integrative Review. Rev. Gen. Psychol..

[28] Koval P., Hollenstein T., Haigh M., Kuppens P. (2023). Emotion Regulation and the Temporal Dynamics of Emotions: Effects of Cognitive Reappraisal and Expressive Suppression on Emotional Inertia. Cogn. Emot..

[29] Walker L.O., Murry T.J. (2022). The Influences of Stress and Health Dynamics in the Postpartum Period. J. Obstet. Gynecol. Neonatal Nurs..

[30] Alves E., Cecatti J.G., Souza J.P. (2021). The Resilience and Health Outcomes in the Postpartum Period: A Systematic Review. J. Psychosom. Obstet. Gynecol..

[31] Bonanno G.A. (2004). Loss, trauma, and human resilience: Have we underestimated the human capacity to thrive after extremely aversive events?. Am. Psychol..

[32] Butler E.A., Lee T.L., Gross J.J. (2007). Emotion regulation and culture: Are the social consequences of emotion suppression culture-specific?. Emotion.

[33] Matsumoto D., Yoo S.H., Nakagawa S. (2008). Culture, emotion regulation, and adjustment. J. Personal. Soc. Psychol..

[34] Hofstede G. (2001). Culture’s Consequences: Comparing Values, Behaviors, Institutions, and Organizations Across Nations.

[35] Collins N.L., Dunkel-Schetter C., Lobel M., Scrimshaw S.C. (1993). Social Support in Pregnancy: Psychosocial Correlates of Birth Outcomes and Postpartum Depression. J. Pers. Soc. Psychol..

[36] Cheadle A.C.D., Ramos M., Schetter C.D. (2020). The Effect of Social Support on Psychological Well-Being in Pregnant Women and Postpartum Mothers. Health Psychol..

[37] Qi X., Yang M., Ren W., Jia J., Wang L., Han G., Fan R. (2022). The Effects of Social Support on Sleep Quality of Medical Staff Treating Patients with Coronavirus Disease 2019 (COVID-19) in January and February 2020 in China. Med. Sci. Monit..

[38] Lakey B., Orehek E. (2011). Relational Regulation Theory: A New Approach to Explain the Link Between Perceived Social Support and Mental Health. Psychol. Rev..

[39] Pearlin L.I., Menaghan E.G., Lieberman M.A., Mullan J.T. (1981). The Stress Process. J. Health Soc. Behav..

